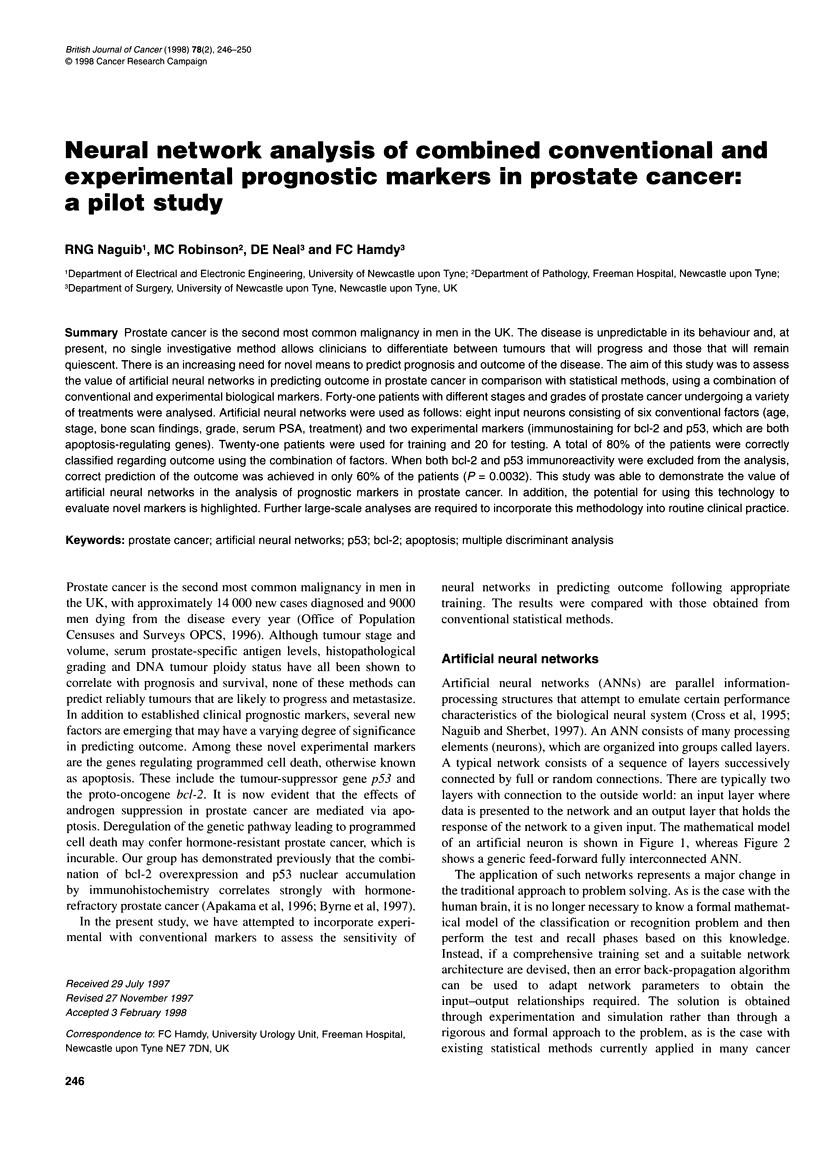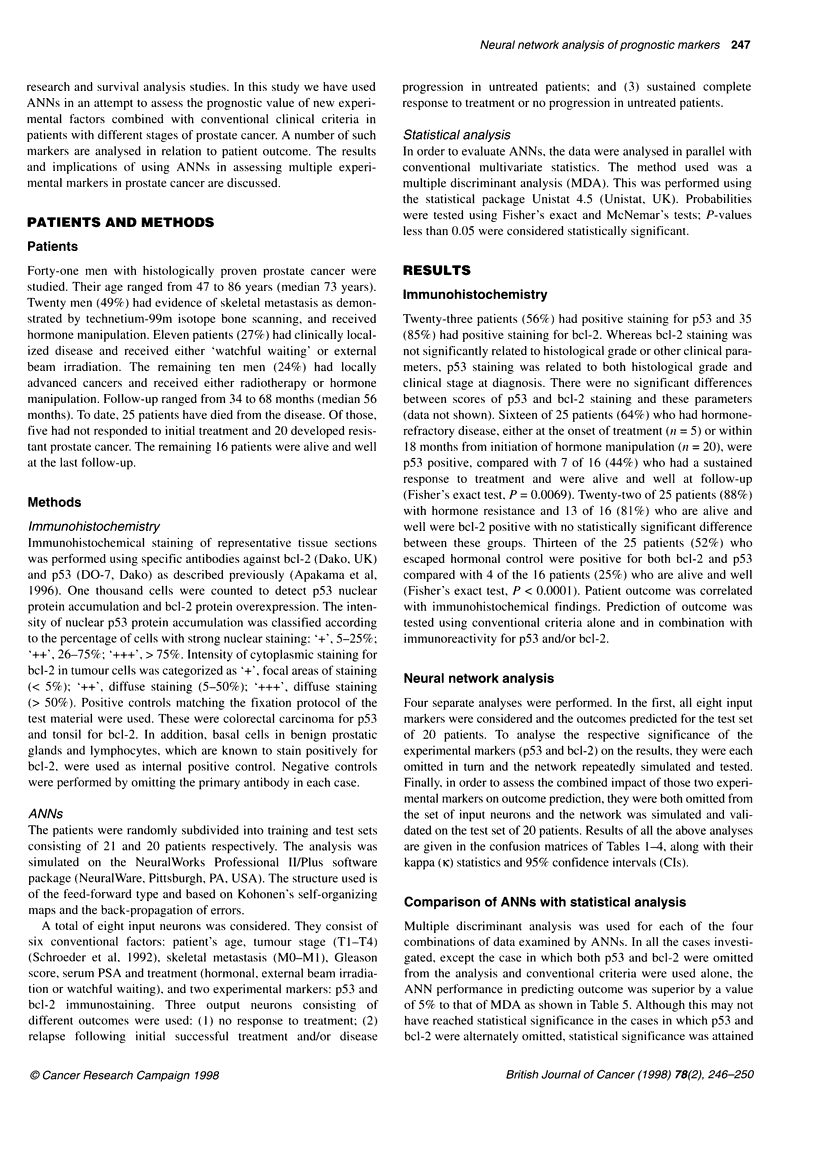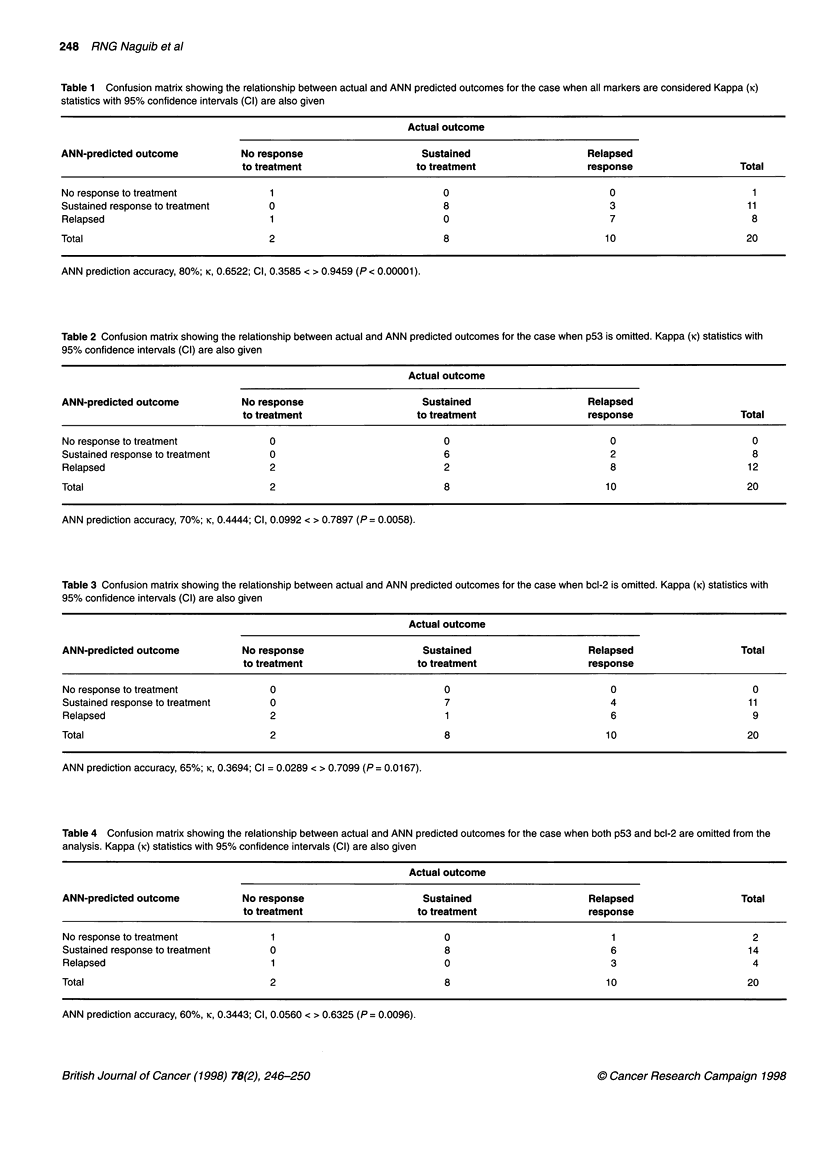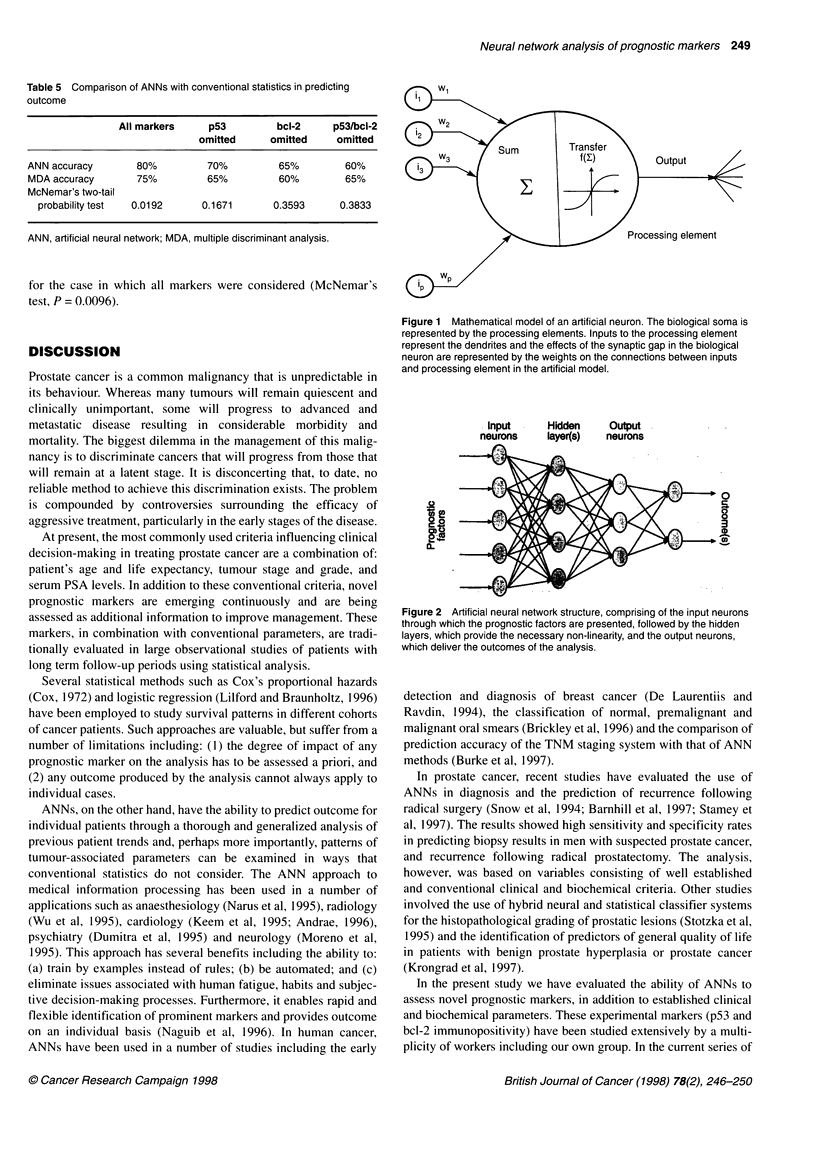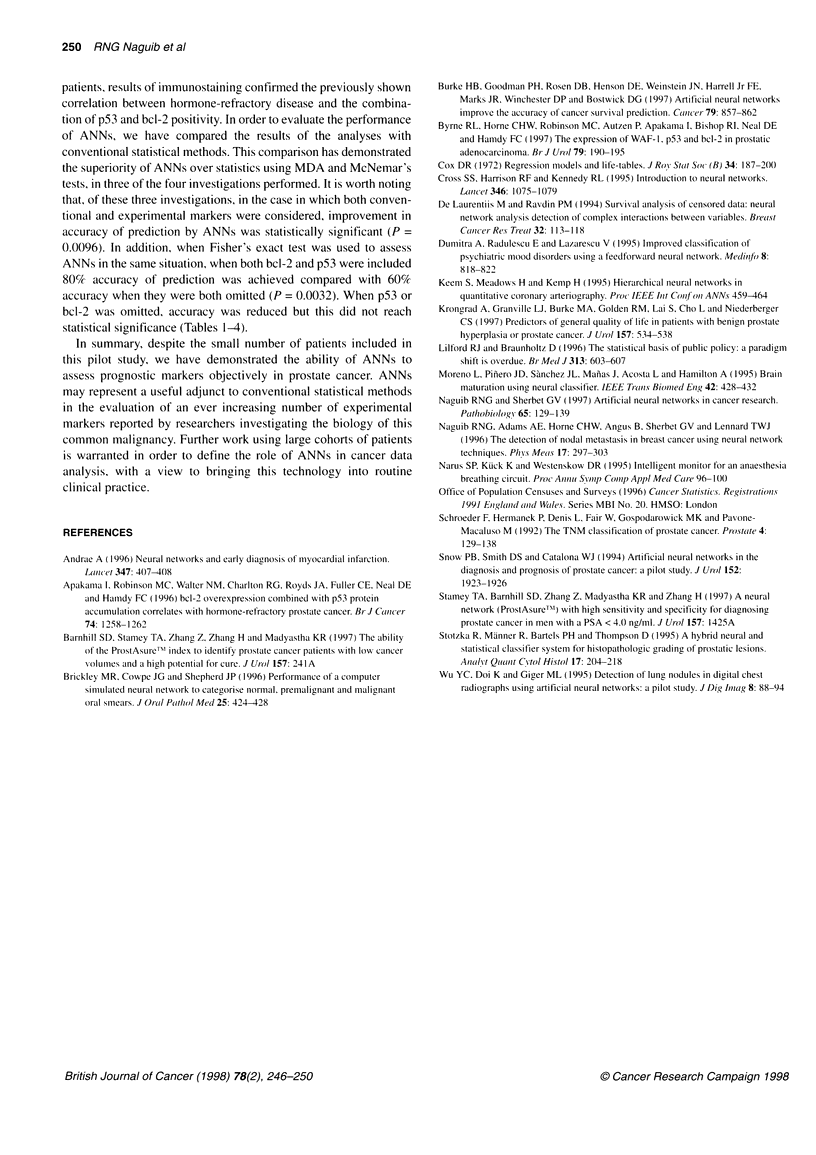# Neural network analysis of combined conventional and experimental prognostic markers in prostate cancer: a pilot study.

**DOI:** 10.1038/bjc.1998.472

**Published:** 1998-07

**Authors:** R. N. Naguib, M. C. Robinson, D. E. Neal, F. C. Hamdy

**Affiliations:** Department of Electrical and Electronic Engineering, University of Newcastle upon Tyne, UK.

## Abstract

**Images:**


British Journal of Cancer (1998) 78(2), 246–250
© 1998 Cancer Research Campaign

### Neural network analysis of combined conventional and experimental prognostic markers in prostate cancer: a pilot study

**RNG Naguib[1], MC Robinson[2], DE Neal[3] and FC Hamdy[3]**

[1]Department of Electrical and Electronic Engineering, University of Newcastle upon Tyne; [2]Department of Pathology, Freeman Hospital, Newcastle upon Tyne; [3]Department of Surgery, University of Newcastle upon Tyne, Newcastle upon Tyne, UK

**Summary** Prostate cancer is the second most common malignancy in men in the UK. The disease is unpredictable in its behaviour and, at present, no single investigative method allows clinicians to differentiate between tumours that will progress and those that will remain quiescent. There is an increasing need for novel means to predict prognosis and outcome of the disease. The aim of this study was to assess the value of artificial neural networks in predicting outcome in prostate cancer in comparison with statistical methods, using a combination of conventional and experimental biological markers. Forty-one patients with different stages and grades of prostate cancer undergoing a variety of treatments were analysed. Artificial neural networks were used as follows: eight input neurons consisting of six conventional factors (age, stage, bone scan findings, grade, serum PSA, treatment) and two experimental markers (immunostaining for bcl-2 and p53, which are both apoptosis-regulating genes). Twenty-one patients were used for training and 20 for testing. A total of 80% of the patients were correctly classified regarding outcome using the combination of factors. When both bcl-2 and p53 immunoreactivity were excluded from the analysis, correct prediction of the outcome was achieved in only 60% of the patients ($P = 0.0032$). This study was able to demonstrate the value of artificial neural networks in the analysis of prognostic markers in prostate cancer. In addition, the potential for using this technology to evaluate novel markers is highlighted. Further large-scale analyses are required to incorporate this methodology into routine clinical practice.

**Keywords:** prostate cancer; artificial neural networks; p53; bcl-2; apoptosis; multiple discriminant analysis

Prostate cancer is the second most common malignancy in men in the UK, with approximately 14 000 new cases diagnosed and 9000 men dying from the disease every year (Office of Population Censuses and Surveys OPCS, 1996). Although tumour stage and volume, serum prostate-specific antigen levels, histopathological grading and DNA tumour ploidy status have all been shown to correlate with prognosis and survival, none of these methods can predict reliably tumours that are likely to progress and metastasize. In addition to established clinical prognostic markers, several new factors are emerging that may have a varying degree of significance in predicting outcome. Among these novel experimental markers are the genes regulating programmed cell death, otherwise known as apoptosis. These include the tumour-suppressor gene *p53* and the proto-oncogene *bcl-2*. It is now evident that the effects of androgen suppression in prostate cancer are mediated via apoptosis. Deregulation of the genetic pathway leading to programmed cell death may confer hormone-resistant prostate cancer, which is incurable. Our group has demonstrated previously that the combination of bcl-2 overexpression and p53 nuclear accumulation by immunohistochemistry correlates strongly with hormone-refractory prostate cancer (Apakama et al, 1996; Byrne et al, 1997).

In the present study, we have attempted to incorporate experimental with conventional markers to assess the sensitivity of neural networks in predicting outcome following appropriate training. The results were compared with those obtained from conventional statistical methods.

*Received 29 July 1997*
*Revised 27 November 1997*
*Accepted 3 February 1998*

*Correspondence to*: FC Hamdy, University Urology Unit, Freeman Hospital, Newcastle upon Tyne NE7 7DN, UK

### Artificial neural networks

Artificial neural networks (ANNs) are parallel information-processing structures that attempt to emulate certain performance characteristics of the biological neural system (Cross et al, 1995; Naguib and Sherbet, 1997). An ANN consists of many processing elements (neurons), which are organized into groups called layers. A typical network consists of a sequence of layers successively connected by full or random connections. There are typically two layers with connection to the outside world: an input layer where data is presented to the network and an output layer that holds the response of the network to a given input. The mathematical model of an artificial neuron is shown in Figure 1, whereas Figure 2 shows a generic feed-forward fully interconnected ANN.

The application of such networks represents a major change in the traditional approach to problem solving. As is the case with the human brain, it is no longer necessary to know a formal mathematical model of the classification or recognition problem and then perform the test and recall phases based on this knowledge. Instead, if a comprehensive training set and a suitable network architecture are devised, then an error back-propagation algorithm can be used to adapt network parameters to obtain the input–output relationships required. The solution is obtained through experimentation and simulation rather than through a rigorous and formal approach to the problem, as is the case with existing statistical methods currently applied in many cancer research and survival analysis studies. In this study we have used ANNs in an attempt to assess the prognostic value of new experimental factors combined with conventional clinical criteria in patients with different stages of prostate cancer. A number of such markers are analysed in relation to patient outcome. The results and implications of using ANNs in assessing multiple experimental markers in prostate cancer are discussed.

### PATIENTS AND METHODS

### Patients

Forty-one men with histologically proven prostate cancer were studied. Their age ranged from 47 to 86 years (median 73 years). Twenty men (49%) had evidence of skeletal metastasis as demonstrated by technetium-99m isotope bone scanning, and received hormone manipulation. Eleven patients (27%) had clinically localized disease and received either 'watchful waiting' or external beam irradiation. The remaining ten men (24%) had locally advanced cancers and received either radiotherapy or hormone manipulation. Follow-up ranged from 34 to 68 months (median 56 months). To date, 25 patients have died from the disease. Of those, five had not responded to initial treatment and 20 developed resistant prostate cancer. The remaining 16 patients were alive and well at the last follow-up.

### Methods

#### *Immunohistochemistry*

Immunohistochemical staining of representative tissue sections was performed using specific antibodies against bcl-2 (Dako, UK) and p53 (DO-7, Dako) as described previously (Apakama et al, 1996). One thousand cells were counted to detect p53 nuclear protein accumulation and bcl-2 protein overexpression. The intensity of nuclear p53 protein accumulation was classified according to the percentage of cells with strong nuclear staining: '+', 5–25%; '++', 26–75%; '+++', > 75%. Intensity of cytoplasmic staining for bcl-2 in tumour cells was categorized as '+', focal areas of staining (< 5%); '++', diffuse staining (5–50%); '+++', diffuse staining (> 50%). Positive controls matching the fixation protocol of the test material were used. These were colorectal carcinoma for p53 and tonsil for bcl-2. In addition, basal cells in benign prostatic glands and lymphocytes, which are known to stain positively for bcl-2, were used as internal positive control. Negative controls were performed by omitting the primary antibody in each case.

#### *ANNs*

The patients were randomly subdivided into training and test sets consisting of 21 and 20 patients respectively. The analysis was simulated on the NeuralWorks Professional II/Plus software package (NeuralWare, Pittsburgh, PA, USA). The structure used is of the feed-forward type and based on Kohonen's self-organizing maps and the back-propagation of errors.

A total of eight input neurons was considered. They consist of six conventional factors: patient's age, tumour stage (T1–T4) (Schroeder et al, 1992), skeletal metastasis (M0–M1), Gleason score, serum PSA and treatment (hormonal, external beam irradiation or watchful waiting), and two experimental markers: p53 and bcl-2 immunostaining. Three output neurons consisting of different outcomes were used: (1) no response to treatment; (2) relapse following initial successful treatment and/or disease progression in untreated patients; and (3) sustained complete response to treatment or no progression in untreated patients.

#### *Statistical analysis*

In order to evaluate ANNs, the data were analysed in parallel with conventional multivariate statistics. The method used was a multiple discriminant analysis (MDA). This was performed using the statistical package Unistat 4.5 (Unistat, UK). Probabilities were tested using Fisher's exact and McNemar's tests; *P*-values less than 0.05 were considered statistically significant.

### RESULTS

### Immunohistochemistry

Twenty-three patients (56%) had positive staining for p53 and 35 (85%) had positive staining for bcl-2. Whereas bcl-2 staining was not significantly related to histological grade or other clinical parameters, p53 staining was related to both histological grade and clinical stage at diagnosis. There were no significant differences between scores of p53 and bcl-2 staining and these parameters (data not shown). Sixteen of 25 patients (64%) who had hormone-refractory disease, either at the onset of treatment ($n = 5$) or within 18 months from initiation of hormone manipulation ($n = 20$), were p53 positive, compared with 7 of 16 (44%) who had a sustained response to treatment and were alive and well at follow-up (Fisher's exact test, $P = 0.0069$). Twenty-two of 25 patients (88%) with hormone resistance and 13 of 16 (81%) who are alive and well were bcl-2 positive with no statistically significant difference between these groups. Thirteen of the 25 patients (52%) who escaped hormonal control were positive for both bcl-2 and p53 compared with 4 of the 16 patients (25%) who are alive and well (Fisher's exact test, $P < 0.0001$). Patient outcome was correlated with immunohistochemical findings. Prediction of outcome was tested using conventional criteria alone and in combination with immunoreactivity for p53 and/or bcl-2.

### Neural network analysis

Four separate analyses were performed. In the first, all eight input markers were considered and the outcomes predicted for the test set of 20 patients. To analyse the respective significance of the experimental markers (p53 and bcl-2) on the results, they were each omitted in turn and the network repeatedly simulated and tested. Finally, in order to assess the combined impact of those two experimental markers on outcome prediction, they were both omitted from the set of input neurons and the network was simulated and validated on the test set of 20 patients. Results of all the above analyses are given in the confusion matrices of Tables 1–4, along with their kappa ($\kappa$) statistics and 95% confidence intervals (CIs).

### Comparison of ANNs with statistical analysis

Multiple discriminant analysis was used for each of the four combinations of data examined by ANNs. In all the cases investigated, except the case in which both p53 and bcl-2 were omitted from the analysis and conventional criteria were used alone, the ANN performance in predicting outcome was superior by a value of 5% to that of MDA as shown in Table 5. Although this may not have reached statistical significance in the cases in which p53 and bcl-2 were alternately omitted, statistical significance was attained

© Cancer Research Campaign 1998

**Table 1** Confusion matrix showing the relationship between actual and ANN predicted outcomes for the case when all markers are considered Kappa (κ) statistics with 95% confidence intervals (CI) are also given

| ANN-predicted outcome | Actual outcome | | | |
|---|---|---|---|---|
| | No response to treatment | Sustained to treatment | Relapsed response | Total |
| No response to treatment | 1 | 0 | 0 | 1 |
| Sustained response to treatment | 0 | 8 | 3 | 11 |
| Relapsed | 1 | 0 | 7 | 8 |
| Total | 2 | 8 | 10 | 20 |

ANN prediction accuracy, 80%; κ, 0.6522; CI, 0.3585 < > 0.9459 ($P < 0.00001$).

**Table 2** Confusion matrix showing the relationship between actual and ANN predicted outcomes for the case when p53 is omitted. Kappa (κ) statistics with 95% confidence intervals (CI) are also given

| ANN-predicted outcome | Actual outcome | | | |
|---|---|---|---|---|
| | No response to treatment | Sustained to treatment | Relapsed response | Total |
| No response to treatment | 0 | 0 | 0 | 0 |
| Sustained response to treatment | 0 | 6 | 2 | 8 |
| Relapsed | 2 | 2 | 8 | 12 |
| Total | 2 | 8 | 10 | 20 |

ANN prediction accuracy, 70%; κ, 0.4444; CI, 0.0992 < > 0.7897 ($P = 0.0058$).

**Table 3** Confusion matrix showing the relationship between actual and ANN predicted outcomes for the case when bcl-2 is omitted. Kappa (κ) statistics with 95% confidence intervals (CI) are also given

| ANN-predicted outcome | Actual outcome | | | |
|---|---|---|---|---|
| | No response to treatment | Sustained to treatment | Relapsed response | Total |
| No response to treatment | 0 | 0 | 0 | 0 |
| Sustained response to treatment | 0 | 7 | 4 | 11 |
| Relapsed | 2 | 1 | 6 | 9 |
| Total | 2 | 8 | 10 | 20 |

ANN prediction accuracy, 65%; κ, 0.3694; CI = 0.0289 < > 0.7099 ($P = 0.0167$).

**Table 4** Confusion matrix showing the relationship between actual and ANN predicted outcomes for the case when both p53 and bcl-2 are omitted from the analysis. Kappa (κ) statistics with 95% confidence intervals (CI) are also given

| ANN-predicted outcome | Actual outcome | | | |
|---|---|---|---|---|
| | No response to treatment | Sustained to treatment | Relapsed response | Total |
| No response to treatment | 1 | 0 | 1 | 2 |
| Sustained response to treatment | 0 | 8 | 6 | 14 |
| Relapsed | 1 | 0 | 3 | 4 |
| Total | 2 | 8 | 10 | 20 |

ANN prediction accuracy, 60%, κ, 0.3443; CI, 0.0560 < > 0.6325 ($P = 0.0096$).

 © Cancer Research Campaign 1998

**Table 5** Comparison of ANNs with conventional statistics in predicting outcome

| | All markers | p53 omitted | bcl-2 omitted | p53/bcl-2 omitted |
|---|---|---|---|---|
| ANN accuracy | 80% | 70% | 65% | 60% |
| MDA accuracy | 75% | 65% | 60% | 65% |
| McNemar's two-tail probability test | 0.0192 | 0.1671 | 0.3593 | 0.3833 |

ANN, artificial neural network; MDA, multiple discriminant analysis.

for the case in which all markers were considered (McNemar's test, $P = 0.0096$).

### DISCUSSION

Prostate cancer is a common malignancy that is unpredictable in its behaviour. Whereas many tumours will remain quiescent and clinically unimportant, some will progress to advanced and metastatic disease resulting in considerable morbidity and mortality. The biggest dilemma in the management of this malignancy is to discriminate cancers that will progress from those that will remain at a latent stage. It is disconcerting that, to date, no reliable method to achieve this discrimination exists. The problem is compounded by controversies surrounding the efficacy of aggressive treatment, particularly in the early stages of the disease.

At present, the most commonly used criteria influencing clinical decision-making in treating prostate cancer are a combination of: patient's age and life expectancy, tumour stage and grade, and serum PSA levels. In addition to these conventional criteria, novel prognostic markers are emerging continuously and are being assessed as additional information to improve management. These markers, in combination with conventional parameters, are traditionally evaluated in large observational studies of patients with long term follow-up periods using statistical analysis.

Several statistical methods such as Cox's proportional hazards (Cox, 1972) and logistic regression (Lilford and Braunholtz, 1996) have been employed to study survival patterns in different cohorts of cancer patients. Such approaches are valuable, but suffer from a number of limitations including: (1) the degree of impact of any prognostic marker on the analysis has to be assessed a priori, and (2) any outcome produced by the analysis cannot always apply to individual cases.

ANNs, on the other hand, have the ability to predict outcome for individual patients through a thorough and generalized analysis of previous patient trends and, perhaps more importantly, patterns of tumour-associated parameters can be examined in ways that conventional statistics do not consider. The ANN approach to medical information processing has been used in a number of applications such as anaesthesiology (Narus et al, 1995), radiology (Wu et al, 1995), cardiology (Keem et al, 1995; Andrae, 1996), psychiatry (Dumitra et al, 1995) and neurology (Moreno et al, 1995). This approach has several benefits including the ability to: (a) train by examples instead of rules; (b) be automated; and (c) eliminate issues associated with human fatigue, habits and subjective decision-making processes. Furthermore, it enables rapid and flexible identification of prominent markers and provides outcome on an individual basis (Naguib et al, 1996). In human cancer, ANNs have been used in a number of studies including the early

**Figure 1** Mathematical model of an artificial neuron. The biological soma is represented by the processing elements. Inputs to the processing element represent the dendrites and the effects of the synaptic gap in the biological neuron are represented by the weights on the connections between inputs and processing element in the artificial model.

**Figure 2** Artificial neural network structure, comprising of the input neurons through which the prognostic factors are presented, followed by the hidden layers, which provide the necessary non-linearity, and the output neurons, which deliver the outcomes of the analysis.

detection and diagnosis of breast cancer (De Laurentiis and Ravdin, 1994), the classification of normal, premalignant and malignant oral smears (Brickley et al, 1996) and the comparison of prediction accuracy of the TNM staging system with that of ANN methods (Burke et al, 1997).

In prostate cancer, recent studies have evaluated the use of ANNs in diagnosis and the prediction of recurrence following radical surgery (Snow et al, 1994; Barnhill et al, 1997; Stamey et al, 1997). The results showed high sensitivity and specificity rates in predicting biopsy results in men with suspected prostate cancer, and recurrence following radical prostatectomy. The analysis, however, was based on variables consisting of well established and conventional clinical and biochemical criteria. Other studies involved the use of hybrid neural and statistical classifier systems for the histopathological grading of prostatic lesions (Stotzka et al, 1995) and the identification of predictors of general quality of life in patients with benign prostate hyperplasia or prostate cancer (Krongrad et al, 1997).

In the present study we have evaluated the ability of ANNs to assess novel prognostic markers, in addition to established clinical and biochemical parameters. These experimental markers (p53 and bcl-2 immunopositivity) have been studied extensively by a multiplicity of workers including our own group. In the current series of

© Cancer Research Campaign 1998

patients, results of immunostaining confirmed the previously shown correlation between hormone-refractory disease and the combination of p53 and bcl-2 positivity. In order to evaluate the performance of ANNs, we have compared the results of the analyses with conventional statistical methods. This comparison has demonstrated the superiority of ANNs over statistics using MDA and McNemar's tests, in three of the four investigations performed. It is worth noting that, of these three investigations, in the case in which both conventional and experimental markers were considered, improvement in accuracy of prediction by ANNs was statistically significant ($P$ = 0.0096). In addition, when Fisher's exact test was used to assess ANNs in the same situation, when both bcl-2 and p53 were included 80% accuracy of prediction was achieved compared with 60% accuracy when they were both omitted ($P$ = 0.0032). When p53 or bcl-2 was omitted, accuracy was reduced but this did not reach statistical significance (Tables 1–4).

In summary, despite the small number of patients included in this pilot study, we have demonstrated the ability of ANNs to assess prognostic markers objectively in prostate cancer. ANNs may represent a useful adjunct to conventional statistical methods in the evaluation of an ever increasing number of experimental markers reported by researchers investigating the biology of this common malignancy. Further work using large cohorts of patients is warranted in order to define the role of ANNs in cancer data analysis, with a view to bringing this technology into routine clinical practice.

### REFERENCES

Andrae A (1996) Neural networks and early diagnosis of myocardial infarction. *Lancet* **347**: 407–408

Apakama I, Robinson MC, Walter NM, Charlton RG, Royds JA, Fuller CE, Neal DE and Hamdy FC (1996) bcl-2 overexpression combined with p53 protein accumulation correlates with hormone-refractory prostate cancer. *Br J Cancer* **74**: 1258–1262

Barnhill SD, Stamey TA, Zhang Z, Zhang H and Madyastha KR (1997) The ability of the ProstAsure™ index to identify prostate cancer patients with low cancer volumes and a high potential for cure. *J Urol* **157**: 241A

Brickley MR, Cowpe JG and Shepherd JP (1996) Performance of a computer simulated neural network to categorise normal, premalignant and malignant oral smears. *J Oral Pathol Med* **25**: 424–428

Burke HB, Goodman PH, Rosen DB, Henson DE, Weinstein JN, Harrell Jr FE, Marks JR, Winchester DP and Bostwick DG (1997) Artificial neural networks improve the accuracy of cancer survival prediction. *Cancer* **79**: 857–862

Byrne RL, Horne CHW, Robinson MC, Autzen P, Apakama I, Bishop RI, Neal DE and Hamdy FC (1997) The expression of WAF-1, p53 and bcl-2 in prostatic adenocarcinoma. *Br J Urol* **79**: 190–195

Cox DR (1972) Regression models and life-tables. *J Roy Stat Soc (B)* **34**: 187–200

Cross SS, Harrison RF and Kennedy RL (1995) Introduction to neural networks. *Lancet* **346**: 1075–1079

De Laurentiis M and Ravdin PM (1994) Survival analysis of censored data: neural network analysis detection of complex interactions between variables. *Breast Cancer Res Treat* **32**: 113–118

Dumitra A, Radulescu E and Lazarescu V (1995) Improved classification of psychiatric mood disorders using a feedforward neural network. *Medinfo* **8**: 818–822

Keem S, Meadows H and Kemp H (1995) Hierarchical neural networks in quantitative coronary arteriography. *Proc IEEE Int Conf on ANNs* 459–464

Krongrad A, Granville LJ, Burke MA, Golden RM, Lai S, Cho L and Niederberger CS (1997) Predictors of general quality of life in patients with benign prostate hyperplasia or prostate cancer. *J Urol* **157**: 534–538

Lilford RJ and Braunholtz D (1996) The statistical basis of public policy: a paradigm shift is overdue. *Br Med J* **313**: 603–607

Moreno L, Piñero JD, Sànchez JL, Mañas J, Acosta L and Hamilton A (1995) Brain maturation using neural classifier. *IEEE Trans Biomed Eng* **42**: 428–432

Naguib RNG and Sherbet GV (1997) Artificial neural networks in cancer research. *Pathobiology* **65**: 129–139

Naguib RNG, Adams AE, Horne CHW, Angus B, Sherbet GV and Lennard TWJ (1996) The detection of nodal metastasis in breast cancer using neural network techniques. *Phys Meas* **17**: 297–303

Narus SP, Kück K and Westenskow DR (1995) Intelligent monitor for an anaesthesia breathing circuit. *Proc Annu Symp Comp Appl Med Care* 96–100

Office of Population Censuses and Surveys (1996) *Cancer Statistics. Registrations 1991 England and Wales*. Series MBI No. 20. HMSO: London

Schroeder F, Hermanek P, Denis L, Fair W, Gospodarowick MK and Pavone-Macaluso M (1992) The TNM classification of prostate cancer. *Prostate* **4**: 129–138

Snow PB, Smith DS and Catalona WJ (1994) Artificial neural networks in the diagnosis and prognosis of prostate cancer: a pilot study. *J Urol* **152**: 1923–1926

Stamey TA, Barnhill SD, Zhang Z, Madyastha KR and Zhang H (1997) A neural network (ProstAsure™) with high sensitivity and specificity for diagnosing prostate cancer in men with a PSA < 4.0 ng/ml. *J Urol* **157**: 1425A

Stotzka R, Männer R, Bartels PH and Thompson D (1995) A hybrid neural and statistical classifier system for histopathologic grading of prostatic lesions. *Analyt Quant Cytol Histol* **17**: 204–218

Wu YC, Doi K and Giger ML (1995) Detection of lung nodules in digital chest radiographs using artificial neural networks: a pilot study. *J Dig Imag* **8**: 88–94

© Cancer Research Campaign 1998